# Antioxidant Stress of Transdermal Gene Delivery by Non-Viral Gene Vectors Based on Chitosan-Oligosaccharide

**DOI:** 10.3390/jfb13040299

**Published:** 2022-12-15

**Authors:** Pengfei Cui, Ting Zhu, Pengju Jiang, Jianhao Wang

**Affiliations:** School of Pharmacy, Changzhou University, Changzhou 213164, China

**Keywords:** chitosan-oligosaccharide, non-viral vector, transdermal, ROS, gene delivery

## Abstract

Oxidative stress initiated by reactive oxygen species (ROS) is the cause of many acquired or congenital skin diseases. Oral antioxidants or using topical antioxidants preparations may bring the nonspecific distribution of drugs or anaphylaxis due to repeated use. In this study, a biocompatible gene vector by cross-linking of chitosan-oligosaccharide (CSO) and N,N’-cystamine-bis-acrylamide (CBA) was synthesized (CSO-CBA), which could carry therapeutic genes into the skin, express functional proteins in epidermal cells, and play an efficient antioxidant effect. Infrared and ^1^H NMR spectrum data showed that CSO-CBA was successfully synthesized. Gel electrophoresis results showed that the gene could be successfully compressed by the carrier and can be released in a reducing environment. Hemolysis experiments showed that the carrier had good biocompatibility. Transdermal gene delivery experiments proved that the vector can bring genes into the skin, express functional proteins, and protect the skin from reactive oxygen species damage after 7 days of administration. Skin compatibility experiments show that our therapy is biocompatible. Our study provides a minimally invasive and painless, high-biocompatibility, and long-term effective treatment for skin damage caused by reactive oxygen species, which has a potential application.

## 1. Introduction

Skin is the body’s first physiological barrier against external damage [1]. Ultraviolet radiation, microbial infection, radiation therapy, environmental pollution, aging, and congenital diseases, as exogenous or endogenous sources of reactive oxygen species (ROS), may lead to the deterioration of skin function, inducing erythema, wrinkles, photoaging, autoimmune reactions, hypersensitivity, keratosis, precancerous lesions, and skin cancer [2,3,4,5]. ROS are a class of oxygen-free radicals containing or capable of producing unpaired electrons 4. Biomolecular damage caused by elevated ROS levels is characterized by lipid peroxidation, enzyme inactivation/activation, DNA mutation/fragmentation, and protein oxidation/degradation [6]. We can fight against oxidative stress by oral or topical application of antioxidants, including vitamin C, vitamin E, glutathione (GSH), curcumin, and β-carotene [7,8,9]. However, the efficacy of small-molecule antioxidants is usually dose-dependent and requires multiple administrations, causing nonspecific distribution and inconvenience [10,11]. Topical applications may employ penetration enhancers, and frequent application will cause skin sensitivity and discomfort [12].

Gene therapy provides a fundamental solution for the treatment of skin diseases [13,14]. Gene therapy aims to introduce genetic medicines into target cells, thereby promising to cure diseases that cannot be cured by traditional methods [15]. Nowadays, gene therapy has played an important role in tumor immunotherapy and the treatment of congenital diseases, including congenital skin diseases [16,17]. However, gene therapy also faces many risks, such as the systemic safety of viral vectors, and the overexpression of gene therapy will bring some new problems [18,19]. In addition, researchers have also found that gene drugs are easily degraded in the body, and nucleic acids in the free form are rapidly degraded by nucleases present in the extracellular matrix [20]. Therefore, in order to effectively deliver gene drugs to target cells, it is imperative to develop a safe and efficient carrier system. At present, gene delivery vector systems are mainly divided into two categories: viral vectors and non-viral vectors. Non-viral vectors have entered the public field with the advantages of low cost, simple preparation, and good biocompatibility. Non-viral vectors improve the therapeutic effect by enhancing the accommodation of nucleic acid substances and prolonging the circulation period in the body. Chitosan is often used as a non-viral vector.

Chitosan is a class of cationic polymers composed of glucosamine with good biocompatibility and biodegradability [21]. It has been widely used in dermatology applications due to its safety and antibacterial properties [22,23]. As a cationic polymer, chitosan can compress negatively charged genes to form nanoparticles through electrostatic interaction, providing high transfection efficiency in vitro. However, only relying on a simple non-viral vector has limited cargo-carrying capacity and insufficient targeting. The surface of chitosan has abundant functional groups, which can be modified by introducing new ligands to encapsulate or adsorb the target gene, so the sustained release and targeting performance of the delivery system can be improved. Zhang [24] synthesized stimuli-responsive cross-linked chitosan by utilizing the disulfide bonds and acyl hydrazone bonds of chitosan and simultaneously cross-linked PEI and PEG. The complex is capable of dual stimulatory responses in redox and acidic environments to release DNA. Kamra [25] also used the carboxymethylation properties of chitosan-oligosaccharide itself to further derivatize pyridine-substituted products and at the same time replaced nearly 40% of the primary alcohols of chitosan with oxyimino functional groups to synthesize a hydroxylamine derivative. Both derivatives achieved co-delivery of the p53 gene and doxorubicin drug in vitro and in vivo. Liu [26] introduced a new ligand, grafted histidine to chitosan, and self-assembled with siRNA to form an HGCS/siRNA nano-delivery system. The uptake of the system by cells showed that HGCS has high efficiency. The siRNA transfection ability can be demonstrated in B16F1 cells and HeLa cells. All in all, the application of chitosan as a cationic non-viral carrier has been widely developed and utilized and achieved remarkable results. However, from the perspective of gene carrier delivery and release, the particle size of the carrier/DNA system will affect the effect of cellular endocytosis. Jae-Woon Nah [27] investigated the molecular-weight-dependent in vitro and in vivo absorption phenomena of water-soluble chitosan. The absorption of chitosan was significantly influenced by its molecular mass. With the increasing molecular mass, the absorption decreased. Chitosan has a large molecular weight and poor water solubility at physiological pH 7.4. Moreover, the chitosanase that can hydrolyze β-glycosides in the human digestive tract is relatively lacking. Based on the above reasons, researchers turned their attention to chitosan-oligosaccharide [28,29]. Chitosan-oligosaccharide (CSO) is a product of the decomposition of chitosan and has better biocompatibility [30,31]. However, CSO makes it difficult to achieve the protection of genes due to its lower molecular weight.

Herein, we designed a non-viral gene vector based on CSO. Specifically, the CSO was cross-linked to form macromolecule CSO-CBA via a reducible-response linker N,N’-cystamine-bis-acrylamide (CBA) [32]. The vector can effectively protect the gene and exhibits superior cell transfection efficiency and biocompatibility. Catalase (CAT) is an endogenous ROS scavenger with efficient ROS scavenging ability. CAT plasmid was employed in this research to verify the vector effect, and hydrogen peroxide was used as a model to verify whether the CAT gene can effectively resist oxidation. In order to overcome the stratum corneum barrier, we use microneedle-induced pores. Microneedles have the advantages of both subcutaneous injection and percutaneous delivery. When applied to the skin, they can form microcatheters, allowing loaded genes to bypass the stratum corneum and enter the skin microcirculation, thereby achieving gene therapy. Therefore, CSO-CBA-coated therapeutic genes can penetrate deep into the skin, release CAT gene to express CAT protein, and play a strong role in clear ROS.

## 2. Materials and Methods

### 2.1. Materials

Chitosan-oligosaccharides (CSO, 970Da) were obtained from cool chemical science and technology (Beijing, China), and the degree of deacetylation of CSO is 95%. Cystamine dihydrochloride (98%) and acryloyl chloride (97%) were purchased from Bailingwei Technology Company (Beijing, China). Jiangsu Qiangsheng Functional Chemical Company provided dichloromethane (DCM; analytical grade). Lipo8000™ was provided by Beyotime Biotechnology (Shanghai, China). Fetal bovine serum (FBS), trypsin, and penicillin-streptomycin were purchased from Gibco (USA). Dulbecco’s modified eagle’s medium (DMEM) and Lipo8000™ were supplied by Beyotime Biotechnology (Shanghai, China). GFP antibody, CAT plasmid with N terminal GFP Spark tag, and GFP plasmid was obtained from Sino Biological (Beijing, China). The Fourier-transform infrared spectrometer (FT-IR) is from Thermo Fisher Scientific’s Nicolet™ iS50 FTIR Spectrometer series (Massachusetts, USA). The solid microneedles are from Shanghai Fanzhen Trading Co., Ltd. Catalase detection kits, and Western and IP cell lysates, fixatives, and blocking solutions were purchased from Beyotime Biotechnology Co., Ltd. (Shanghai, China). Nanjing Jiancheng Bioengineering Institute provided alanine aminotransferase and aspartate aminotransferase detection kits (Nanjing, China).

### 2.2. Culture Conditions of L929 Cells

L929 cells (Mouse epidermal-derived cells, ATCC, USA) were used with 10% (*v/v*) of fetal bovine serum and 1% (*v/v*) penicillin-streptomycin in Dulbecco’s modified eagle’s medium (DMEM) cell culture medium and cultured in a 5% CO_2_, 37 °C cell incubator.

### 2.3. Synthesis of N,N’-Cystamine-Bis-Acrylamide(CBA)

We followed the known synthetic method of N,N’-cystamine-bis-acrylamide [33]. The aqueous solution of cystamine dihydrochloride and the dichloromethane solution of acryloyl chloride and sodium hydroxide were titrated and mixed in sequence under an ice bath. Then, the mixture was stirred at room temperature for 6 h to obtain crude CBA. Finally, pure CBA was obtained by a series of methods such as dichloromethane extraction, solution rotary evaporation, and precipitate drying. The yield of CBA% was then calculated from the following Equation (1):

(1) $$Yield\;of\;\mathrm{CBA}\left(\%\right)=\frac{\frac{m\left(\mathrm{BCA}\right)}{M\left(\mathrm{BCA}\right)}}{n\left(Cystamine\;Dihydrochloride\right)}\times 100\%\;$$

### 2.4. Synthesis of Chitosan-Oligosaccharide (CSO)- N,N’-Cystamine-Bis-Acrylamide (CBA)

There are abundant functional groups on the surface of chitosan-oligosaccharide, which can encapsulate or adsorb target genes. Its modification sites are mainly primary amines and hydroxyl groups [34]. We choose N,N’-cystamine-bis-acrylamide cross-linking agent and chitosan-oligosaccharide for Michael’s addition reaction to modify primary amines. First, we weighed an appropriate amount of chitosan-oligosaccharide (CSO) and dissolve it with a small amount of anhydrous DMSO at room temperature, then added an appropriate amount of triethylamine and stir evenly. The molar ratio of chitosan-oligosaccharide to triethylamine is 1:3. Next, we took an appropriate amount of N,N’-cystamine-bis-acrylamide and dissolved it in an appropriate amount of anhydrous DMSO at room temperature. (The molar ratio of CSO-CBA ranges from 1:0.2 to 1:1.2.) The DMSO solution of chitosan-oligosaccharide and the DMSO solution of N,N’-cystamine-bis-acrylamide were mixed uniformly at room temperature, transferred to a pressure-resistant bottle, and oil-bathed at 60 °C for 24 h. After adjusting the pH to 4, the solution was then dialyzed with pure water in a dialysis bag with a molecular weight cut-off value of 3500. After lyophilization, the finished product CSO-CBA was obtained, and the structure was analyzed by FT-IR. CBA was dissolved in DMSO, and CSO and CSO-CBA were dissolved in deuterated water for ^1^H-NMR analysis. The yield of CSO-CBA% was then calculated from the following Equation (2):

(2) $$Yield\;of\;\mathrm{CSO}-\mathrm{CBA}\left(\%\right)=\frac{m\left(\mathrm{CSO}-\mathrm{CBA}\right)}{m\left(\mathrm{CSO}\right)+m\left(\mathrm{CBA}\right)}\times 100\%\;$$

### 2.5. Synthesis and Characterization of CSO-CBA/DNA

#### 2.5.1. Size

CSO-CBA was configured into an aqueous solution of 1 mg/mL, and an equal volume of DNA was added dropwise when the carrier solution was in a vortex state. After the DNA was completely added to the carrier solution, the vortex also ended. Thus far, CSO-CBA/DNA gene carrier system nanoparticles were prepared. The particle size distribution of CSO-CBA and CSO-CBA/DNA were determined at room temperature with a Zetasizer (Malvern, UK). In addition, the morphology of CSO-CBA was observed by transmission electron microscopy (TEM).

#### 2.5.2. Agarose Gel Electrophoresis

The nano-forming results of CSO-CBA/DNA were characterized by agarose gel electrophoresis. Agar powder was used to prepare 1% agarose gel, and TAE buffer was used as an electrolyte. CSO-CBA was combined with DNA in five different weight ratios (25, 50, 75, 100, 200, *w/w*). Then, diluent and load color developer were added. The final total volume of DMEM and loading reagent buffer was 12 μL. Finally, the electrophoresis apparatus was powered on at 110 v for 30 min. To test the DNA condensation ability under reducing conditions, the same complexes were prepared and incubated with 5 mg/mL DTT for 4 h at 25 °C before electrophoresis.

### 2.6. Biocompatibility Characterization of CSO-CBA

The normally cultured L929 cells were washed with PBS, digested with trypsin, pelleted by centrifugation at 1500 rpm for 3 min, and then counted using a cytometer. The cells were seeded in a 96-well plate at a density of 8000 cells/mL. Finally, cells were incubated for 12 h at 37 °C, 5% CO_2_. The old medium was then discarded. Subsequent replacements were followed by different concentrations of CSO-CBA samples (0, 10, 20, 30, 50, 100 μg/mL) diluted in a medium. With 1% triton as a positive control, four replicate wells were set for each sample. We incubated again for 24 h under the same incubation conditions, and then, 20 μL of MTT solution was added to each well, and the liquid in the well was discarded after 4 h incubation. Finally, anhydrous DMSO was added, and after 5 min of shaking, the OD_570nm_ was measured using a microplate reader.

Fresh blood was withdrawn from the orbital vein of normal mice and collected with an anticoagulant tube. The erythrocytes were collected by centrifugation at 2000 rpm in a refrigerated centrifuge at 4 °C for 10 min. RBCs were washed three times with 300 μL of pre-chilled PBS, pH 7.4. The final solution was centrifuged at 4 °C, 2000 rpm for 5 min, and the supernatant was discarded again. Then, PBS at pH 7.4 was added to the treated red blood cells to prepare a 20% red blood cell suspension. 1% Triton and PBS (pH 7.4) were used as positive and negative controls, respectively. CSO-CBA was diluted into samples with concentrations of 20, 50, 100, 200, 400, and 800 μg/mL in PBS at pH 7.4. We added 20 μL of 20% red blood cell suspension to each of the prepared 1 mL negative control, positive control, and six concentration gradient samples. Samples were then incubated at 37 °C for 2 h. Finally, these samples were centrifuged at 4 °C, 2000 rpm, for 10 min and photographed. At the same time, red blood cells incubated with 1 mg/mL CSO-CBA, 1mg/mL PEI_25K_, PBS, and 1% TX-100 samples were taken for microscopic imaging.

### 2.7. Screening of Solid Microneedles Delivery Depth

We rotated 42-gauge solid microneedles to different scales (250 μm, 500 μm, 750 μm, 1000 μm, 1250 μm, 1500 μm, 1750 μm, and 2000 μm) and inserted them into 2% agar. Then, 500 μL of Coomassie brilliant blue solution was added to the hole, and the depth of infiltration was observed by microscope after 10 min.

### 2.8. CAT Delivery In Vitro

The normally cultured L929 cells were washed with PBS, digested with trypsin, and then centrifuged at 1500 rpm for 3 min to pellet the cells. The cells were then counted using a cell counter and seeded in a 24-well plate at a density of 10^5^ cells/mL. We incubated the cells for about 16 h in a 37 °C, 5% CO_2_ incubator. The DNA was added to an equal volume of CSO-CBA solution while vortexing. The solution was vortexed and placed for 20 min to replace the original culture medium in the 24-well plate. After a further 4 h of incubation, the cells were replaced with fresh cell culture medium. Each well was photographed 24 h later using an inverted fluorescence microscope, and the CAT in living cells was quantified by flow cytometry.

### 2.9. Quantification of Intracellular Catalase Activity

Normally cultured L929 cells were digested with trypsin. Then, the cells were collected by centrifugation at 1500 rpm for 3 min. Cells were plated in 24-well plates at a cell density of 1×10^5^/mL and incubated at 37 °C in a 5% CO_2_ incubator until the cell density was 70–80%. CSO-CBA/CAT was prepared by mixing 1 μg CAT (catalase) and 200 μg CSO-CBA with it. After 48 h, the original solution was discarded, and 100 μL of Western and IP cell lysate was added to each well to lyse the cells. Then, the cell suspension was centrifuged at 12,000 rpm for 5 min under a refrigerated centrifuge, and the supernatant was taken. Finally, a catalase detection kit was used to determine the amount of catalase activity in the supernatant. At the same time, the blank group (L929 cells) and the positive control group (Lipo8000^TM^/CAT) were set up.

### 2.10. Screening of H_2_O_2_ Concentration

L929 cells were seeded in 96-well plates at a density of 40,000/mL per well and incubated overnight in a 5% CO_2_ incubator at 37 °C. Then, the original medium was replaced with 0–5 μM H_2_O_2_, and 20 μL of MTT was added to each well after 24 h. Co-incubation was performed for 4 h to remove the liquid in the hole, with 150 μL of DMSO added after. Finally, the OD_570nm_ of the plate was measured.

### 2.11. Antioxidant Evaluation In Vitro

The cells were spread in a 24-well plate at a density of 1 ×10^5^/mL. After incubation in a cell incubator overnight, 500 μL of the configured CSO-CBA/CAT solution was added to each well. After another 48 h of incubation, the appropriate H_2_O_2_ concentration was added. Subsequently, MTT was added after 24 h, and DMSO was added after 4 h. Finally, the OD_570nm_ of the plate was measured.

At the same time, in another 24-well plate, the supernatant was taken out after 24 h without adding MTT, and the remaining H_2_O_2_ concentration in the supernatant was measured to verify the antioxidant effect.

### 2.12. Solid Microneedles Delivery of CAT In Vivo

CSO-CBA/CAT was inserted into the skin of female BALB/c mice (4–6 weeks) by solid microneedles. At the same time, a blank mouse group was set up, with three mice in each group. After 7 days, the skin tissue was taken, embedded with frozen embedding agent (OCT), and cut into 10 μm slices. After 10 min of fixation, 1 h of blocking, and 1 h of antibody incubation, the sections were placed under a fluorescence inverted microscope to observe the green fluorescent protein.

At the same time, mice were delivered CAT through microneedles. The corresponding skin tissue was taken out and then ground using a tissue homogenizer. The tissue fluid was centrifuged at 8000× *g* for 10 min in a refrigerated centrifuge. Finally, the supernatant was taken for the determination of CAT content. Normal mice were set as the blank group, and mice with microneedle delivery of Lipo8000^TM^/CAT were set as the positive control group, with three mice in each group.

### 2.13. Antioxidation and Safety Evaluation In Vivo

BALB/c mice were randomly divided into six groups (*n* = 3 in each group) as follows: (1) only 20% H_2_O_2_ was applied; (2) solid microneedles were used only, and 20% H_2_O_2_ was applied after 48 h; (3) after CSO-CBA/GFP was delivered by solid microneedles, 20% H_2_O_2_ was applied 48 h later; (4) after Lipo8000^TM^/CAT was delivered by solid microneedles, 20% H_2_O_2_ was applied after 48 h; (5) after CSO-CBA/CAT was delivered by solid microneedles, 20% H_2_O_2_ was applied after 48 h; and (6) normal mice. After 7 days of transdermal administration, the corresponding skin tissues were removed for hematoxylin-eosin staining (H & E).

The orbital blood of the first five groups of mice was taken, and the serum was taken after centrifugation at 2000 rpm for 10 min. Safety tests were performed using alanine aminotransferase and aspartate aminotransferase kits.

### 2.14. Statistical Analysis

All statistical analyses were performed using SPSS Statistics 26.0. The results of experiments were expressed as the mean ± standard deviation (SD, *n* = 3). Statistical comparisons were performed by one-way or two-way analysis of variance (ANOVA) for multiple groups.

## 3. Results and Discussion

### 3.1. Characterization of CSO-CBA Structure

**Figure 1:**
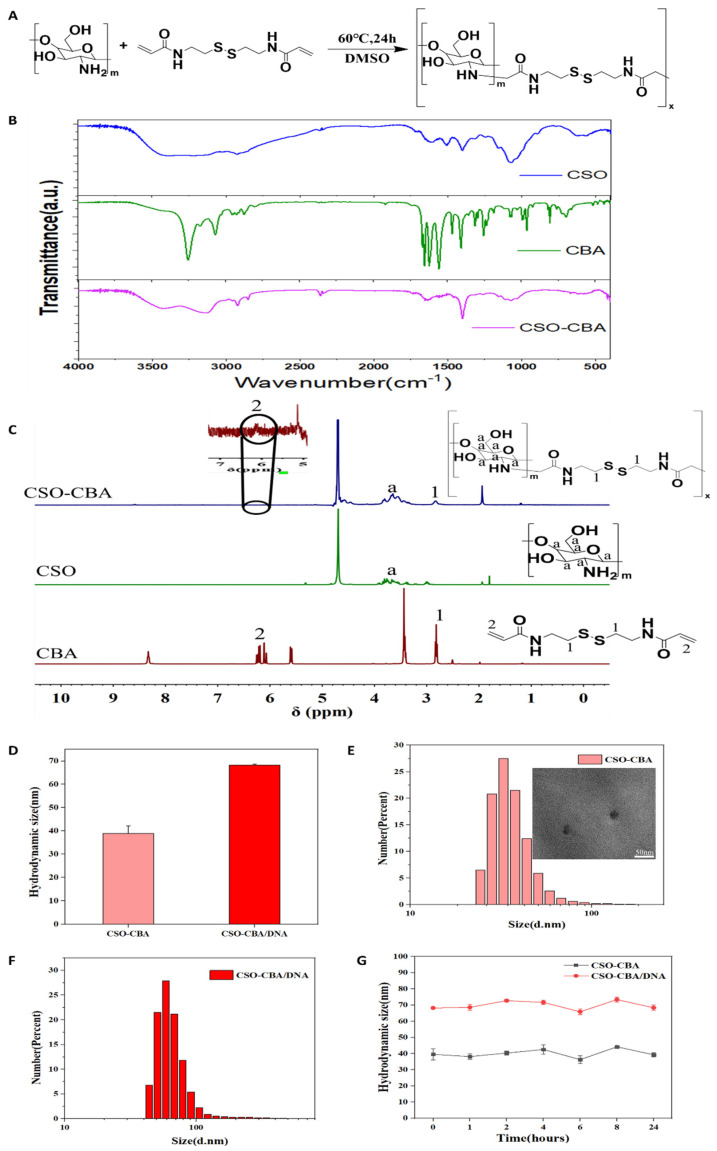
Characterization of CSO-CBA and CSO-CBA/DNA. (**A**) The reaction equation of CSO-CBA. “M” and “X” are positive integers, respectively. (**B**) The Fourier infrared spectrum of CSO, CBA, and CSO-CBA. (**C**) ^1^H-NMR for characterization of CSO-CBA, CBA, and CSO (300 MHz). (**D**) The hydrated particle size of CSO-CBA and CSO-CBA/DNA. (**E**) Particle size distribution of CSO-CBA, and the illustration is the TEM of CSO-CBA. Scale bar, 50 nm. (**F**) Particle size distribution of CSO-CBA/DNA. (**G**) Stability of CSO-CBA and CSO-CBA/DNA for 24 h at 25 °C (*n* = 3, mean ± SD).

N,N’-cystamine-bis-acrylamide is a degradable cross-linking agent that assembles with chitosan oligosaccharides, which can increase the chain length of small-molecule chitosan oligosaccharides. The reaction mechanism is that the primary amine of chitosan oligosaccharide and the carbon–carbon double bond of N,N’-cystamine-bis-acrylamide are combined by Michael addition, and the specific reaction equation is shown in Figure 1A. The structure of the product CSO-CBA was characterized by FT-IR. Figure 1B shows that the characteristic band of the carbon–carbon double bond (1670–1620 cm^−1^) originally belonging to CBA in CSO-CBA disappeared. The broad band of CSO at 3440 cm^−1^ is due to -OH and -NH stretching vibration, and the band at 2880 cm^−1^ belongs to -CH stretching vibration [35]. The amide band appears at 1665 cm^−1^. These bands also appear in CSO-CBA. As shown in Figure 1C, the proton peak position “1” of CBA appears in CSO-CBA, and the obvious proton peak position “a” of CSO also appears in CSO-CBA. The position “2” represents signals attributed to H-atoms of CSO linked with C = C of CBA. In the enlarged image, it can be seen that almost all the ends of each CBA are connected to the CSO. The above confirmed that we successfully synthesized CSO-CBA. Figure 1D–F show the particle size and distribution of CSO-CBA and CSO-CBA/DNA, indicating that the prepared nanocomposites are all well-dispersed systems, with concentrated distributions of 39 nm and 68 nm, respectively. The TEM results in Figure 1E show that the size of CSO-CBA is 32 nm, which is consistent with the results of particle size In addition, the particle size stability of CSO-CBA and CSO-CBA/DNA within 24 h was also investigated. The results show that the aqueous solutions of the two nanoparticles can be stably dispersed at 25 °C (Figure 1G). In general, we synthesized well-dispersed and stable CSO-CBA nanoparticles.

### 3.2. Characterization of CSO-CBA/DNA Self-Assembled Nano-Systems

**Figure 2:**
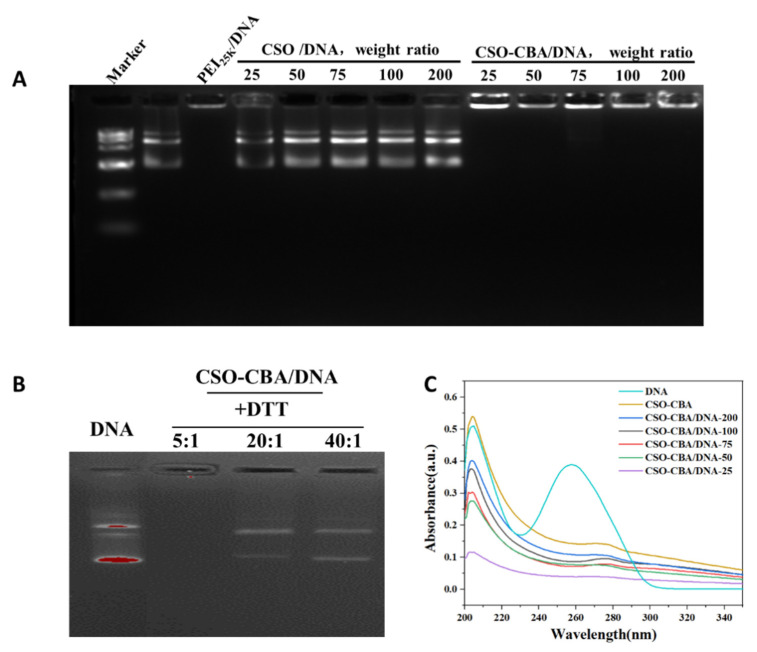
Agarose gel electrophoresis of CSO-CBA series and DNA. (**A**) Agarose gel electrophoresis images of different weight ratios (25/50/75/100/200) of CSO and CBA when the molar ratio of CSO and CBA is 1:1.2. (**B**) Agarose gel electrophoresis images of CSO-CBA/DNA co-incubated with DTT at different weight ratios. (**C**) Purple absorption spectra of DNA, CSO-CBA, and different weight ratios of CSO-CBA/DNA (25/50/75/100/200).

Whether the gene carrier can be effectively combined with DNA is the primary condition for gene transfection [36]. Agarose gel electrophoresis has the dual functions of “molecular sieve” and “electrophoresis”. The negatively charged DNA runs from the positive electrode to the negative electrode under electrophoresis, and there are obvious bands. It can be seen from Figure 2A that the PEI_25K_ vector in the control group can bind to DNA and stay in the sample well, while the CSO/DNA in the experimental group will have a band, indicating that CSO and DNA alone cannot self-assemble. When the molar ratio of CSO: CBA is 1:1.2 (represented by CSO-CBA-1.2 in the figure), it can bind DNA well at different weight ratios (25/50/75/100/200:1). This aspect also verifies the successful synthesis of CSO-CBA under this molar ratio. At the same time, we also verified other different molar ratios of CSO and CBA to bind to DNA. The results were not as good as CSO-CBA-1.2, so the product with a molar ratio of CSO to CBA of 1:1.2 (referred to as CSO-CBA) was used in the following experiments. In order to verify the DNA loading capacity of the system, we scanned the UV absorption spectra of DNA, CSO-CBA, and CSO-CBA/DNA with different mass ratios. It can be seen from Figure 2C that the individual DNA UV absorption spectrum has a specific peak position at 260 nm, while other samples do not show this characteristic. It indicated that DNA was loaded into different ratios of CSO-CBA. DTT (dithiothreitol) is a powerful reducing agent [37] that cleaves disulfide bonds to induce DNA release in CSO-CBA/DNA. The biodegradability of CSO-CBA under reducing conditions was examined using DTT. Figure 2B shows that when the weight ratio of CSO-CBA/DNA to DTT reaches more than 20:1, DNA can be released well.

### 3.3. Characterization of CSO-CBA Biocompatibility

**Figure 3:**
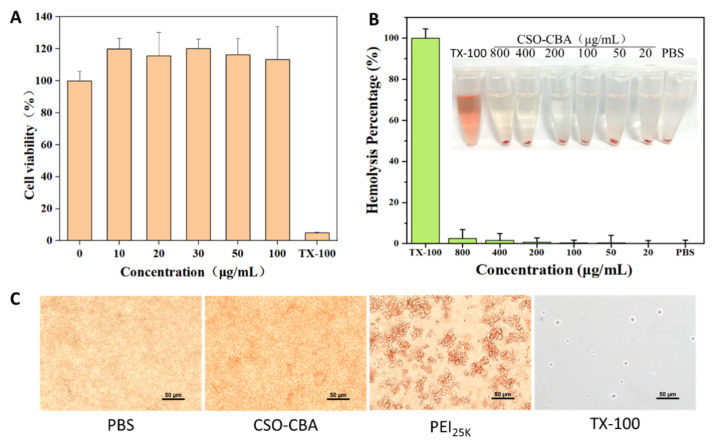
Results of CSO-CBA biocompatibility. (**A**) Cytotoxicity test results of different concentrations of CSO-CBA (0, 10, 20, 30, 50, 100 μg/mL) in L929 cells. (**B**) The results of hemolysis experiments with different concentrations of CSO-CBA (20, 50, 100, 200, 400, 800 μg/mL). Data were presented as mean ± s.d. (*n* = 3). (**C**) Microscopic imaging of red blood cells incubated with 1mg/mL CSO-CBA, 1mg/mL PEI_25K_, PBS, and 1% TX-100 samples. Scale bar, 50 μm.

Chitosan-oligosaccharide is a cationic polymer with good biocompatibility and biodegradability, while N,N’-cystamine-bis-acrylamide is a degradable disulfide. We used mouse epidermal-derived cells L929 cells to perform cytotoxicity experiments on the synthesized complex CSO-CBA. Compared with the positive control TX-100 and the negative control DMEM, different concentrations of CSO-CBA were not toxic to cells, as shown in Figure 3A. The cell morphology was intact, and the number increased, and the cell viability was close to 100%. This may be because chitosan oligosaccharide is a degradation product of chitin and has been reported to have a wide range of physiological functions and biological activities. CSO mainly plays a role in repairing cell surface sugar chains and can promote protein synthesis and cell activation [38]. Secondly, the orbital blood of Balb/c mice was used for the hemolysis experiment of CSO-CBA. The picture of Figure 3B shows that when the concentration of CSO-CBA reaches 800 μg/mL, the red blood cells still do not rupture and dissolve. Compared with the negative control PBS, the hemolysis rate of all concentrations of TX-100 was less than 5%. In addition, the morphology of red blood cells in 1 mg/mL CSO-CBA was not different from that of the negative control PBS (Figure 3C). All of the above show that the biocompatibility of CSO-CBA is good.

### 3.4. CSO-CBA Delivers CAT In Vitro

**Figure 4:**
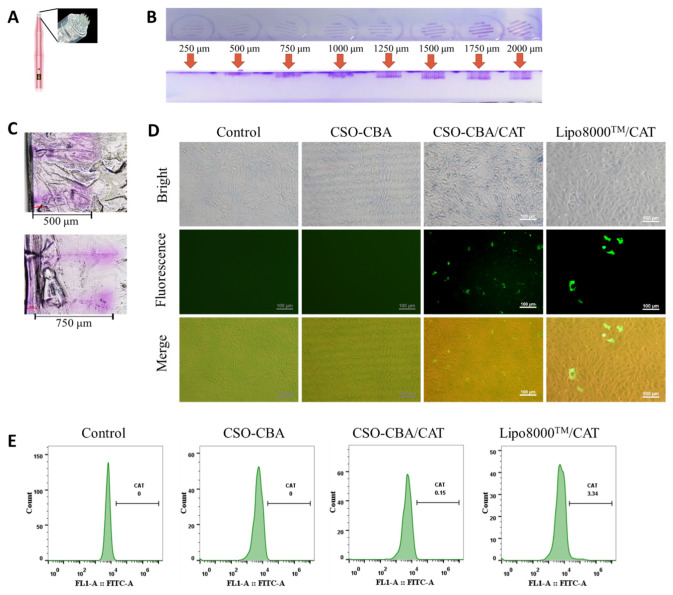
Effect of CSO-CBA on CAT delivery in vitro by (**A**) 42-needle solid microneedles. (**B**) The depth corresponds to different scales of microneedles. (**C**) Inverted microscope steel needle of 500 μm and 750 μm, corresponding to the depth. The results of L929 cells transfection under an inverted fluorescence microscope (**D**) and the results of fluorescent molecule quantification by flow cytometry (**E**). DMEM, CSO-CBA, and Lipo8000^TM^ were blank control, negative control, and positive control, respectively. Scale bar, 100 μm.

Microneedles are a minimally invasive and painless gene drug delivery method, usually between 10 μm and 1000 μm in length. Figure 4A–C show the result of verifying the length of the steel needle. As the microneedles scale increases, the penetration of Coomassie brilliant blue dye becomes deeper (Figure 4B). Combined with the thickness of the epidermis on the back of the mouse, we chose the size of 750 μm.

After determining the length of the microneedles, it was time to select the corresponding skin fibroblasts and verify the effect of the CSO-CBA vector to deliver the gene. Transfection is a technique in which exogenous genes are introduced into cells by a vector material. The number of genes successfully introduced can directly reflect the targeting level and delivery efficiency of the vector. The CAT plasmid we used has a GFP tag in the end; therefore, the delivery efficiency of CAT can be detected by fluorescence microscope and flow cytometry. The transfection results in Figure 4D show that CSO-CBA could deliver the CAT gene into L929 cells. In addition, by detecting the amount of fluorescence in living cells by flow cytometry, we can see that at the cell level, the amount of CAT delivered by CSO-CBA reached 0.15%, while Lipo8000^TM^ delivery was 3.34% (Figure 4E). These results demonstrated that CSO-CBA could successfully deliver genes into mouse cells.

### 3.5. Antioxidant Effect of CAT In Vitro

**Figure 5:**
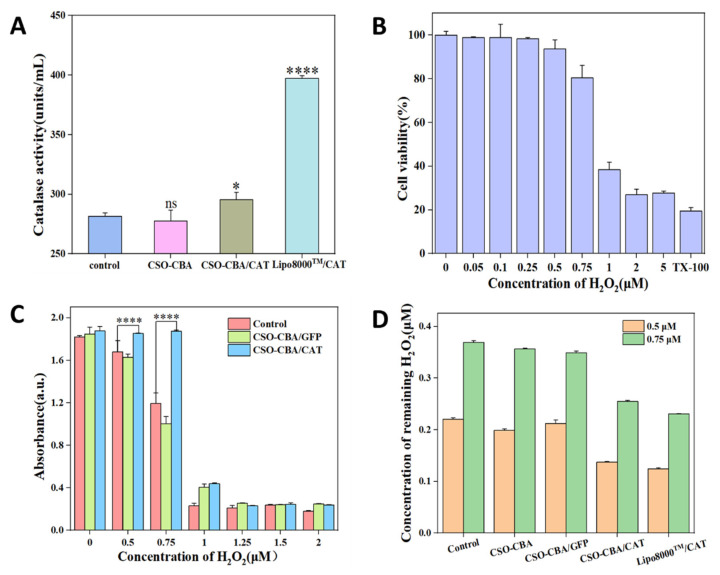
Antioxidant effect of CSO-CBA/CAT in vitro. (**A**) Determination results of intracellular catalase content corresponding to control, CSO-CBA, CSO-CBA/CAT, and Lipo8000^TM^/CAT. (**B**) The effect of 0–5 μM H_2_O_2_ concentration on L929 cell viability, with TX-100 as a positive control. (**C**) The viability of L929 cells was measured after adding 0–2 μM H_2_O_2_ to the blank group, GFP group, and CAT group. (**D**) The content of residual H_2_O_2_ in the supernatant of each group at the concentration of 0.5 μM and 0.75 μM. The significance of the samples and controls was tested by using SPSS software to analyze the obtained *p*-values. ns, *p* > 0.05; *, *p* < 0.05; ****, *p* < 0.0001.

CAT is an endogenous enzyme. In order to evaluate the necessity of delivering CAT genes to cells, the original catalase content of cells was detected. The effects of CSO-CBA and Lipo8000^TM^ on CAT delivery were also quantitatively observed. As shown in Figure 5A, the catalase content detected after CSO-CBA delivery of CAT was significantly different from that of the blank group.

Antioxidation is an active oxygen that resists damage to the body. Active oxygen refers to active free radicals, and hydrogen peroxide is one of them. Therefore, we used hydrogen peroxide as a model to verify whether the CSO-CBA/CAT can effectively resist oxidation. In order to deliver the CAT gene to cells and highlight the effect of scavenging H_2_O_2_, it is necessary to screen out the optimal range of H_2_O_2_ concentration. Finally, on this basis, the antioxidant effect of CAT in vitro was studied. Here, L929 cells were used to detect the cytotoxicity of H_2_O_2_ in the concentration range of 0–5 μM (Figure 5B). We selected 0.5–2 μM H_2_O_2_, in which the cells were both viable for gene transfection and able to detect whether H_2_O_2_ could be effectively removed after adding CAT. It can be found in Figure 5C that when 0.5 μM and 0.75 μM H_2_O_2_ were added after the delivery of the CAT gene, the cell viability of L929 was higher than that without the CAT gene. In addition, by verifying the remaining H_2_O_2_ content of the supernatant at the same concentration, we can also see that the remaining H_2_O_2_ content of the CAT group is indeed less than that of other groups (Figure 5D). The above can prove that at the cellular level, the delivery of the CAT gene can effectively remove H_2_O_2_ so as to achieve the effect of antioxidants.

### 3.6. Solid Microneedles Delivery of CAT In Vivo

**Figure 6:**
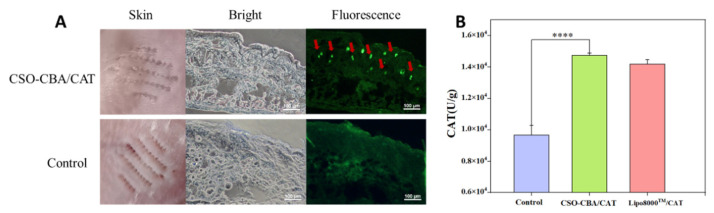
The effect of solid microneedles delivery CSO-CBA/CAT in vivo. (**A**) The visible and fluorescent images of mouse skin were obtained after MNs were applied to the control group and CSO-CBA/CAT group, respectively. Scale bar, 100 μm. (**B**) CAT content in skin tissue corresponding to control, CAO-CBA/CAT, and Lipo8000^TM^/CAT. The significance of the samples and controls was tested by using SPSS software to analyze the obtained *p*-values. ****, *p* < 0.0001.

Therefore, the effect of transdermal delivering CSO-CBA/CAT via solid microneedles in vivo was tested. The skin of the mice was obtained after 7 days of administration. The fluorescence wavelength of GFP protein is short, and it is easy to be masked by the background fluorescence of the tissue, so the expression of GFP in the skin sections was determined by GFP antibody-mediated immunofluorescence staining. As shown in Figure 6A, compared with the control group, there were obvious green highlights on the fluorescence imaging of CSO-CBA (red arrows), indicating that solid microneedles could deliver CAT to mouse skin. At the same time, we homogenized the skin of the mouse, extracted catalase, and determined the content. It can be found that the content of CAT in the skin of the CSO-CBA/CAT group reached 14750 U/g, which was significantly higher than that of the blank group at 9668 U/g (Figure 6B). Interestingly, although the transfection efficiency of CSO-CBA was not as good as Lipo8000^TM^ in vitro (Figure 4E), in vivo results of CSO-CBA showed higher transfection efficiency than Lipo8000^TM^, and this may be due to the fact that polymer carriers maintain better stability in vivo than liposomes. In addition, after 7 days of gene delivery, the expression of functional proteins remained at a relatively high level, indicating that they could exert antioxidant activity for a long time, which also avoided the inconvenience of repeated administration or irritation to the skin.

### 3.7. Antioxidant Effect and Safety Evaluation of CSO-CBA/CAT In Vivo

**Figure 7:**
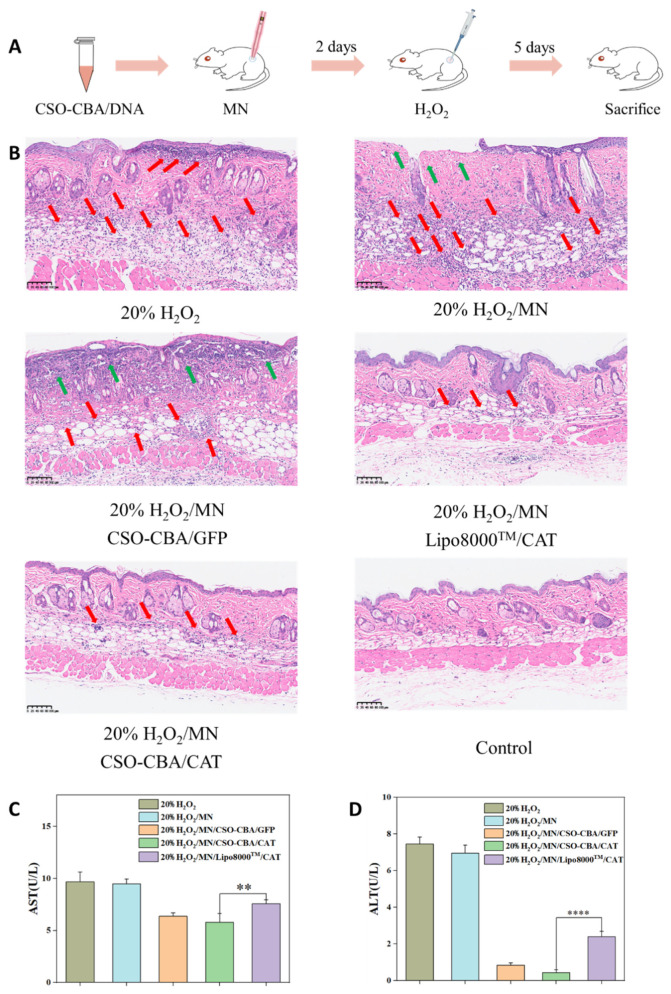
Antioxidant effect and safety evaluation of CSO-CBA/CAT in vivo. (**A**) Flow chart of antioxidant experiment in BALB/c mice. (**B**) The skin H&E results of 20% H_2_O_2_ group, 20% H_2_O_2_/MN group, 20% H_2_O_2_/MN CSO-CBA/GFP group, 20% H_2_O_2_/MN Lipo8000^TM^/CAT group, 20% H_2_O_2_/MN CSO-CBA/CAT group, and control group. Scale bar, 100μm. AST (**C**) and ALT (**D**) results in the blood of mice in 20% H_2_O_2_ group, 20% H_2_O_2_/MN group, 20% H_2_O_2_/MN CSO-CBA/GFP group, 20% H_2_O_2_/MN Lipo8000^TM^/CAT group, and 20% H_2_O_2_/MN CSO-CBA/CAT group. The significance of the samples and controls was tested by using SPSS software to analyze the obtained *p*-values. **, *p* < 0.01; ****, *p* < 0.0001.

In further, the morphological changes of BALB/c mice skin were observed by hematoxylin-eosin (H&E) staining to evaluate the antioxidant activity of CSO-CBA/CAT. GFP plasmid, which does not have the ability to scavenge oxidation, was employed here to act as a negative control. As shown in Figure 7, CSO-CBA/GFP, CSO-CBA/CAT, and Lipo8000^TM^/CAT were delivered to the back of mice by microneedles, and then, 20% H_2_O_2_ was applied. After 7 days of transdermal administration, the skin tissues were collected. We observed that compared with the control group and the only 20% H_2_O_2_/MN group, the CSO-CBA/CAT group had an intact epidermis, compact structure, and less inflammatory cell infiltration (red arrows represent inflammatory cell infiltration, and green arrows represent injured skin structures), which was consistent with the previous content determination (Figure 6B). At last, the safety of percutaneous gene therapy on animals was also evaluated. Alanine aminotransferase (ALT) and aspartate aminotransferase (AST) are present in a variety of cells, primarily hepatocytes. When liver function is severely impaired, enzymes in the cells are released into the blood, resulting in abnormally elevated alanine aminotransferase and aspartate aminotransferase. Therefore, ALT and AST are sensitive markers of acute liver cell damage. We evaluated the safety of our CSO-CBA vector by measuring the ALT and AST levels in the serum of the above groups of mice. The normal values of AST and ALT are 0–40 U/L. The higher the value, the more serious the liver damage. According to Figure 7C, the AST and AST values of the CSO-CBA/CAT group were lower than those of the other groups, including Lipo8000^TM^/CAT group, indicating that our carrier material of CSO-CBA demonstrates biocompatibility. 

## 4. Conclusions

We synthesized a non-viral gene carrier CSO-CBA by cross-linking degradable CBA with chitosan oligosaccharide with good biocompatibility. Through FT-IR and H-NMR characterization, it was found that the carbon–carbon double bond of CBA was added to the primary amine of CSO, and the carrier CSO-CBA was successfully obtained. The gene carrier has a simple preparation method, good biocompatibility, and stability in combination with DNA. Under reducing conditions, the CSO-CBA/DNA nano-system can completely release DNA due to the breakage of the complex disulfide bonds. We also verified the ability of the CSO-CBA vector to deliver DNA into mouse cells. Finally, catalase genes were employed. The results of in vivo antioxidant experiments showed that the transdermal delivery of CSO-CBA could effectively deliver genes to mouse epidermal cells and express the functional protein CAT, providing better protection for skin damaged by reactive oxygen species. Furthermore, after 7 days of gene delivery, the expression of functional proteins remained relatively high, indicating that they could exert antioxidant activity for an extended period, avoiding the inconvenience of repeated administration or skin irritation. Our study provides a minimally invasive and painless, high-biocompatibility, and long-term effective treatment for skin damage caused by reactive oxygen species.
